# A glucose tolerant *β*-glucosidase from a newly isolated *Neofusicoccum parvum* strain F7: production, purification, and characterization

**DOI:** 10.1038/s41598-023-32353-6

**Published:** 2023-03-29

**Authors:** Nivisti Singh, Bruce Sithole, Ajit Kumar, Roshini Govinden

**Affiliations:** 1 Discipline of Microbiology, School of Life Sciences, Westville Campus, University of KwaZulu-Natal, Durban, South Africa; 2 Discipline of Engineering, Howard Campus, University of KwaZulu-Natal, Durban, South Africa; 3 Biorefinery Industry Development Facility, Council for Scientific and Industrial Research, Durban, South Africa

**Keywords:** Biotechnology, Microbiology

## Abstract

Cellulase-producing microorganisms produce low titres of *β*-glucosidases with low tolerance to glucose. This study aimed to improve production, purify, and characterize a *β*-glucosidase from a newly isolated *Neofusicoccum parvum* strain F7. *β*-Glucosidase production was significantly enhanced by a sequential statistical modelling approach from 1.5-fold in Plackett–Burman design to 2.5 U/ml in the Box–Behnken design compared to the preliminary one variable at a time experiments (1.6 U/ml). The optimal conditions for enzyme production by BBD were 12 days of fermentation at 20 °C, 175 rpm, 0.5% glycerol and 1.5% casein in pH 6.0 buffer. Three *β*-glucosidase isoforms referred to as Bgl1, Bgl2, Bgl3 were purified and characterized from the optimized crude extract displaying IC_50_ values of 2.6, 22.6 and 319.5 mM for glucose, respectively. Bgl3 with a molecular mass of approximately 65 kDa demonstrated the highest tolerance to glucose among the isoforms. The optimum activity and stability for Bgl3 was observed at pH 4.0 in 50 mM sodium acetate buffer with 80% *β*-glucosidase residual activity retained for three hours. This isoform also retained 60% residual activity at 65 °C for one hour which was then reduced to 40% which remained stable for another 90 min. The *β*-glucosidase activity of Bgl3 was not enhanced after the addition of metal ions in assay buffers. The *K*_m_ and *v*_max_ for *4*-nitrophenyl-*β*-d-glucopyranoside were 1.18 mM and 28.08 µmol/min, respectively indicating high affinity for the substrate. The ability to withstand the presence of glucose in conjunction with its thermophilic nature indicates promise for this enzyme in industrial application.

## Introduction

*β*-Glucosidases (EC 3.2.1.21) act synergistically with endoglucanase (EC 3.2.1.4) and exoglucanase (3.2.19) to hydrolyse cellulose to glucose monomers^1^. The action of *β*-glucosidases is the rate-limiting step as *β*-glucosidases are often inhibited by their product glucose resulting in feedback inhibition therefore glucose tolerant *β*-glucosidases are required for efficient hydrolysis of cellulose to glucose monomers^2^. *β*-Glucosidases are classified based on three characteristics (i) substrate specificity, (ii) nucleotide sequence, and (iii) amino acid sequence^3^. Based on substrate specificity *β*-glucosidases are classified into three groups aryl-*β*-d-glucosidases which have a strong affinity for aryl-*β*-d-glucosides such as *4*-nitrophenyl-*β*-d-glucopyranoside; cellobiases that hydrolyse only disaccharides; and broad specificity glucosidases that exhibit activity on many substrate types and are the most common^4^. Classification by nucleotide sequence includes *β*-glucosidases and phospho-*β*-glucosidases from bacteria and mammals (BGA); and *β*-glucosidases from yeasts, moulds, and rumen bacteria (BGB)^4^. The third classification group includes *β*-glucosidases with structural similarity and conserved amino acid sequence motifs^5^.

Currently, there are 133 glycoside hydrolase (GH) families in the Carbohydrate Active enZYme database which are further subdivided into clans based on the similarity of their catalytic domain structures and amino acids based on common ancestry^6^. Sixty-two *β*-glucosidases originating from archaebacteria, plants and animals belong to GH family one and 44 originating from bacteria fungi and yeast belong to GH family three, however, these enzymes may also be found in GH families five, nine, 13 and 116^7^. *β*-Glucosidases can be intracellular, extracellular, or cell-bound enzymes. The GH three family *β*-glucosidases are extracellular, or cell bound whilst those from GH family one are predominantly intracellular^3^. GH one and three family *β*-glucosidases are known to display tolerance to glucose^6^.

Numerous microbes including bacteria, fungi and actinomycetes are ubiquitous in nature and the endo and exogenous microbial enzymes from many of these organisms have been widely explored^8–11^. Specifically, *β*-glucosidases from several fungal species including *Aspergillus*, *Fusarium*, and *Trichoderma* have been explored for glucose-tolerant *β*-glucosidases^9,11,12^. *β*-Glucosidases from *Aspergillus* and *Trichoderma* sp. have been commercialised and are currently used in industrial applications including cellulose hydrolysis, however, their commercial preparations are a huge cost factor for industries^3^. Therefore, there is a need to search for a novel thermophilic, glucose tolerant *β*-glucosidase producer able to withstand basic to slightly acidic environments.

Both the bacterial and fungal enzyme producers studied produce several *β*-glucosidases, however, these enzymes do not display tolerance to glucose due to feedback inhibition at high glucose concentrations^13^. Two methods may be used to produce microbial *β*-glucosidases: solid-state fermentation and submerged fermentation. Submerged fermentation has an advantage which includes shorter fermentation periods for enzyme production^14^. *β*-Glucosidase production is influenced by medium composition and culture conditions. Physical and chemical parameters known to influence *β*-glucosidase culture conditions include incubation time, pH, incubation temperature, agitation speed, nitrogen, and carbon sources^1^. Temperature and pH are the most important factors governing microbial growth. Carbon and nitrogen source supplementation provides an enriched environment for microbial growth thus increasing enzyme production^15^. Screening and optimization of growth conditions are crucial to ensure maximal enzyme production with the potential to reduce *β*-glucosidase production costs^16^.

Optimization of growth conditions can be done via two approaches the classical one variable at a time (OVAT) and statistical Plackett–Burman (PPD) and Box–Behnken design (BBD)^10^. The OVAT technique allows for the optimization of one factor at a time, however, the disadvantage of this method is that it is laborious, time-consuming and does not allow one to study the interaction of variables thus making it impossible to detect the true optimum when multiple different variables come together^17^. Therefore, statistical methods such as PBD and BBD are used to eliminate the limitations of the OVAT optimization process^18^. PBD is a screening technique used to screen media components in shake flasks reducing the total number of experiments, thus determining the most important factors^19^. Response surface methodology using a BBD is an effective method to evaluate the interactions between variables by assessing the effect of independent variables on enzyme production^20,21^.

Cellulose hydrolysis by current commercial cellulase cocktails has been achieved, however, these cocktails require supplementation of *β*-glucosidases as the cocktails do not contain sufficient *β*-glucosidases for the complete hydrolysis of cellulose to glucose^23^. *β*-Glucosidases present in these cocktails are also inhibited by their product glucose thus reducing yields^24^. Current commercial *β*-glucosidases from *T. reesei* are very expensive due to high production costs and, it is, therefore, necessary to search for a native fungal producer of glucose-tolerant *β*-glucosidases for supplementation of existing cellulase cocktails. Therefore, to meet industrial demand, there is a need to optimize the production of enzymes by optimization of growth parameters to produce high levels of *β*-glucosidases. There are various reports on the optimization of growth parameters using PBD and BBD to increase *β*-glucosidase yields^17,25,26^. *β*-Glucosidase production from *Aspergillus terreus* strain EMOO 6–4 and *Paecilomyces variotii* was increased by optimization using the two statistical methods mentioned above^17,27^.

Although there were various reports on the production and characterization of *β*-glucosidases from multiple fungal species there are very few reports on glucose-tolerant *β*-glucosidases. Therefore, the present study optimized glucose-tolerant *β*-glucosidase production by PBD and BBD by a novel glucose-tolerant *β*-glucosidase producer *Neofusicoccum parvum* strain F7. We also report the purification of the crude extract and characterization of the purified glucose tolerant *β*-glucosidase.

## Materials and methods

### Growth of fungal strain

The *Neofusicoccum parvum* strain F7 (*N. parvum* F7) was isolated from the tree bark of *Jugla regia* and identified by NCBI blast in a previous study by Singh et al.^28^. The crude extract contained glucose tolerant *β*-glucosidases; therefore, this isolate was selected for optimization of enzyme production. The fungal culture was plated onto potato dextrose agar and incubated at 30 °C for 5 days until fungal growth was observed. The culture was maintained in 25% glycerol stocks at − 80 °C and mineral oil PDA slants stored at 4 °C^29^.

### Production of *β*-glucosidases

Crude extracellular *β*-glucosidases were produced in a submerged fermentation using minimal media that comprised grams per litre (g/l): (1 g) soy peptone, (1.4 g) (NH_4_)_2_SO_4_, (0.3 g) urea, (2 g) KH_2_PO_4_, (0.34 g) CaCl_2_, (0.3 g) MgSO_4_·7H_2_O, (0.005 g) FeSO_4_·7H_2_O, (0.016 g) MnSO_4_·7H_2_O, (0.0014 g) ZnSO_4_·7H_2_O, (0.002 g) CoCl_2_·7H_2_O, and (0.72 g) cellobiose^1^. Each 250 millilitre (ml) flask contained 25 ml of medium and three 5 mm (mm^2^) mycelial plugs of actively growing hyphae and was incubated at 30 °C for seven days at 125 rpm (New Brunswick Scientific, Incubator Shaker series, Innova 44, Germany). After incubation the cell-free supernatant was recovered by centrifuging the medium at 16,837×*g* for 10 min (Eppendorf centrifuge 5418, Germany) and *β*-glucosidase activity was determined using the method described below.

### *β-*Glucosidase assay

*β*-Glucosidase activity was quantified using the method described previously by Kao et al.^1^. The reaction mixture included 10 µl (µl) of enzyme added to 10 µl of 4 mM *4*-nitrophenyl-*β*-d-glucopyranoside (4-NPG) in 50 mM (mM) sodium acetate buffer (pH 5.0) and incubated at 55 °C for 5 min in a water bath and terminated by the addition of 160 µl 1 molar (M) Na_2_CO_3_. The absorbance was measured at 410 nm using a spectrophotometer (Shimadzu UV1800, Japan). One unit of activity was defined as the amount of enzyme needed to release one micromole (µmol) of phenol equivalents per minute at 55 °C. All experiments were triplicated and a standard curve using 4-nitrophenol (4-NP) in 50 mM sodium acetate buffer (pH 5.0) was established^1^. The Beer–Lambert equation was used to calculate enzyme activity:

$${\text{Enzyme}}\,{\text{activity }}\left( {{\text{U}}\,{\text{ml}}} \right) \, = \Delta {\text{AV}}/\varepsilon {\text{tv,}}$$

where A is the Change in absorbance, V is the Total volume of reaction (ml) divided by ε is the Molar extinction co-efficient of *4*-NP (13 700 M^−1^ cm^−1^), t is the reaction time (minutes) and v is the volume of the enzyme (ml).

### Statistical analysis, experimental design, and data analysis

#### Plackett–Burman design (PBD)

In this study, six variables were selected for the PBD. Incubation time (X_1_), pH (X_2_), Incubation temperature (X_3_), Agitation (X_4_), Casein (X_5_) and Glycerol (X_6_) Table 1. A total of twelve experimental runs were carried out for the six variables with each variable represented by a high (+) and low (−) level. To ensure that a significant effect was observed the high and low levels were equidistant from the optimal level and sufficiently far apart from one another. The levels were selected based on the results obtained from a previous study that optimised *β*-glucosidase production from *N. parvum* F7 using one variable at a time experiments^28^. All the experimental runs were carried out in duplicate and an average of the results was reported in Table 2. The PBD was based on the first-order polynomial model Eq. (1):

1 $${\text{Y }} = \beta_{0} \Sigma \beta_{{\text{i}}} X_{{\text{i}}} ,$$

where Y is the response (peak area and retention factor), *β*_0_ is the model intercept, *β*_i_ is the linear coefficient and X_i_ is the level of the independent variable. The results from PBD were analysed using the R Studio software to estimate the significant factors. To evaluate the significance and fit of the regression model p-values and R coefficients were determined using analysis of variance (ANOVA). A Pareto chart of standardised effects was used to represent screened parameters. After the analysis of each variable, those with the highest significance on *β*-glucosidase production were selected for the second level of optimization by Box–Behnken design (BBD) of Response Surface Methodology (RSM).

**Table 1:** Experimental variables and levels used for optimization of *β*-glucosidase production by the *Neofusicccum parvum* strain F7 in the Plackett–Burman design.

Variables	Symbol code	Units	Experimental values	
			Low level (− 1)	High level (+ 1)
Incubation time	X_1_	Days	8	12
pH	X_2_	–	5	7
Incubation temperature	X_3_	°C	20	40
Agitation	X_4_	rpm	150	200
Casein (nitrogen source)	X_5_	%	1.5	2
Glycerol (carbon source)	X_6_	%	0.25	0.75

**Table 2:** Plackett–Burman design matrix with real coded values of six variables to optimize *β*-glucosidase production by the *Neofusicococcum parvum* strain F7.

Run no	Variable level						Enzyme activity (U/ml)	
	Incubation time (days)	pH	Incubation temperature (°C)	Agitation (rpm)	Casein (%)	Glycerol (%)	Observed	Predicted
1	+ (12)	+ (7)	− (20)	+ (200)	+ (2)	+ (0.75)	1.49	1.17
2	+ (12)	− (5)	− (20)	− (150)	+ (2)	+ (0.75)	0.45	0.50
3	− (8)	− (5)	+ (40)	− (150)	+ (2)	+ (0.75)	0.02	0
4	+ (12)	+ (7)	− (20)	− (150)	− (1.5)	+ (0.75)	0.96	1.00
5	+ (12)	+ (7)	+ (40)	− (150)	− (1.5)	− (0.25)	0.02	0.07
6	− (8)	− (5)	− (20)	+ (200)	− (1.5)	+ (0.75)	0.43	0.50
7	− (8)	− (5)	− (20)	− (150)	− (1.5)	− (0.25)	0.23	0.08
8	+ (12)	− (5)	+ (40)	+ (200)	+ (2)	− (0.25)	0.02	0
9	− (8)	+ (7)	− (20)	+ (200)	+ (2)	− (0.25)	0.2	0.50
10	− (8)	+ (7)	+ (40)	− (150)	+ (2)	+ (0.75)	0.02	0.15
11	− (8)	+ (7)	+ (40)	+ (200)	− (1.5)	− (0.25)	0.02	0
12	+ (12)	− (5)	+ (40)	+ (200)	− (1.5)	+ (0.75)	0.03	0.23

Real values: (), coded values: symbolized as + and –.

#### *RSM *using *BBD*

To elucidate the main interactions and the quadratic effects of the three significant variables from the PBD, the BBD was used with replicated centre points (Table 3). R studio was used for statistical analysis and design of experiments^40^. The three most significant variables from PBD, Incubation time (X_1_), Incubation temperature (X_2_) and Glycerol (X_3_) were subjected to a three-level three factor BBD to determine the effect of the three significant independent variables (Table 3). The design consisted of three replicates of the centre point and 16 combinations as shown in Table 4. The average *β*-glucosidase activity was taken as the response (Y). To obtain an empirical model that relates the response to the independent variables a multiple regression analysis of the data was carried out. The second-order polynomial Eq. (2):

2 $${\text{Y}} = \beta_{0} + \Sigma \beta_{{\text{i}}} {\text{X}}_{{\text{i}}} + \Sigma \beta_{{{\text{ii}}}} X_{{\text{i}}}^{2} + \Sigma \beta_{{{\text{ij}}}} X_{{\text{i}}} X_{{\text{j}}} ,$$

where Y is the response (peak area), β_i_, β_ii_ and β_ij_ are the coefficients of the linear, quadratic and interaction terms respectively. X*i* and X*j* are the independent variables. The average of the duplicates was the response for each run. The data were analysed by two-way ANOVA with Tukey’s multiple comparison tests (p ≤ 0.05) using R studio^40^, and 3D response surface and contour plots were produced using ggplot2^30^.

**Table 3:** Experimental codes and levels of independent variables in the Box–Behnken design for optimal *β*-glucosidase production by the *Neofusicoccum parvum* strain F7.

Variables	Symbol code	Experimental values		
		Low (−)	Zero (0)	High (+ 1)
Incubation time (days)	X_1_	8	10	12
Incubation temperature (°C)	X_2_	5	6	7
Glycerol (carbon source) (%)	X_3_	0.25	0.5	0.75

**Table 4:** Box–Behnken design matrix with real coded values of three variables to optimize *β*-glucosidase production by the *Neofusicoccum parvum* strain F7.

Run no	Variable level			Enzyme activity (U/ml)	
	Incubation time (days)	Incubation temperature (°C)	Glycerol (%)	Observed	Predicted
1	− (8)	− (20)	0 (0.5)	0.37	0.81
2	+ (12)	0 (30)	0 (0.5)	2.51	2.07
3	− (8)	+ (40)	0 (0.5)	0.02	-0.69
4	+ (12)	+ (40)	0 (0.5)	0.03	0.56
5	− (8)	0 (30)	− (0.25)	0.55	0.52
6	+ (12)	0 (30)	− (0.25)	1.92	1.78
7	− (8)	0 (40)	+ (0.75)	0.45	0.54
8	+ (12)	+ (40)	+ (0.75)	0.03	1.79
9	0 (10)	− (20)	− (0.25)	1.97	1.72
10	0 (10)	+ (40)	− (0.25)	0.01	0.18
11	0 (10)	− (20)	+ (0.75)	1.57	1.73
12	0 (10)	+ (40)	+ (0.75)	0.02	0.19
13	0 (10)	0 (30)	0 (0.5)	1.14	1.38
14	0 (10)	0 (30)	0 (0.5)	1.18	1.41
15	0 (10)	0 (30)	0 (0.5)	1.48	1.44
16	0 (10)	0 (30)	0 (0.5)	1.31	1.48

Real values: (), coded values: symbolized as +, 0 and – for high, optimal and low, respectively.

### *β*-Glucosidase purification

All purification steps were carried out at 4 °C. The enzyme was fractionated with powdered ammonium sulphate with the following weight/volume (w/v) saturations 10, 20, 30, 40, 50, 60, 70, 80, 90 and 100%^31^. *β*-Glucosidase was precipitated by adding and dissolving the appropriate mass of powdered ammonium sulphate to the crude extract and stirring at 4 °C overnight. The solution was then centrifuged at 14,000×*g*. The pelleted precipitates were resuspended in 50 mM sodium phosphate buffer (pH 6.0) and dialysed against the same buffer overnight at 4 °C with three changes of the dialysis buffer before overnight dialysis. The dialysis buffer was tested for *β*-glucosidase activity to ensure no enzyme was lost during dialysis. The fraction that displayed the highest activity, was concentrated in a 3 kDa cut-off Amicon centrifugal tube. The concentrated fraction was loaded onto an anion exchange column (HiTrap Q FF 5 ml), connected to the AKTA Purifier (AKTA Purifier, GE Healthcare Bioscience, AB75184, Uppsala Sweden). The column was equilibrated with 20 mM Tris-buffer (pH 8.0). The enzyme was eluted using a 0–2 M sodium chloride gradient at a flow rate of 1.5 ml per minute. Enzyme activity for the eluted fractions was determined and those displaying *β*-glucosidase activity were pooled and concentrated.

### Substrate native-PAGE

The concentrated anion exchange fraction was loaded onto a non-denaturing gel (with an 8% resolving and 4% stacking gel which was electrophorized at 90 V at 4 °C for recovery of the active *β*-glucosidase enzyme following which *β*-glucosidase activity was detected by incubating the gel in 50 mM sodium acetate buffer (pH 5.0) at 4 °C for 10 min. After incubation, the gel was stained with a 0.1% esculin and 0.03% FeCl_3_ solution at 55 °C for 10 min and, washed with distilled water to stop the reaction. The three active bands displaying black precipitation indicative of *β*-glucosidase activity were cut out of the gel, ground in a pre-cooled mortar and the enzymes were leached with 50 mM sodium phosphate buffer (pH 6.0) at 4 °C for 12 h. Thereafter the leachate was centrifuged at 4000×*g* for 10 min and the supernatant containing the enzyme was collected, concentrated, and dialysed in the same buffer^11^.

### SDS-PAGE and protein determination

SDS-PAGE was carried out according to the procedure by Laemmli^32^. A 12% polyacrylamide was prepared. Electrophoresis was carried out at 50 V for 3 h and the gel was stained with Coomassie Brilliant Blue. The approximate molecular mass of the protein was determined from the bands that developed on the gel relative to the spectra multicolour broad range molecular mass markers (ThermoScientific, USA). Protein concentration was determined by the Bradford method using Bovine albumin serum as the standard^33^.

### Effect of glucose on *β*-glucosidase activity

To determine the effect of glucose on enzyme activity. The *β*-glucosidase activity was measured in the presence of glucose at different concentrations (0, 50, 100, 250, 500, and 1000 mM) and the IC_50_ values were calculated^9^. All enzyme activities were reported as relative activity to the 100% activity obtained in the absence of glucose. The enzyme displaying the highest tolerance to glucose (highest IC_50_) was taken forward for further characterization.

### Characterization of glucose tolerant *β*-glucosidase

#### Effect of pH

The optimum pH was determined at 55 °C for five minutes in various buffers: sodium acetate (50 mM, pH 3.0–5.0), sodium phosphate (50 mM, pH 6.0–8.0), and Glycine–NaOH (50 mM, pH 9.0–10.0) containing 4 mM *4*-NPG^34^. The pH stability was carried out by pre-incubating the enzyme in sodium acetate buffers (50 mM, pH 3.0 and 5.0) for three hours at 55 °C with aliquots sampled every 30 min. Residual activity was determined using standard assay conditions. The enzyme in optimum pH buffer without incubation served as the control (100% activity).

#### Effect of temperature

The optimum temperature of the enzyme was determined in sodium acetate buffer (50 mM, pH 4.0) containing 4 mM *4*-NPG from 40 to 80 °C with intervals of 5 °C. The stability of the enzyme was determined in 50 mM sodium acetate buffer (pH 4.0) by pre-incubating the enzyme at 65 °C in the absence of *4*-NPG for three hours with aliquots sampled every 30 min. The residual activity was determined at 65 °C for 5 min as per "β-Glucosidase assay" by using the enzyme in an optimum pH buffer without incubation as the control (100% activity).

#### Effect of metal ions

The effect of metal ions on *β*-glucosidase activity was determined. CaCl_2_, CoCl_2_, FeSO_4_, MgSO_4_, MnSO_4_, and ZnSO_4_ at final concentrations of 4 mM were mixed with the enzyme and incubated at room temperature for 1 h^35^. Thereafter, *β*-glucosidase activity was determined using standard assay conditions and reported as relative activity to the control (100% activity) that was not treated with a metal ion.

#### Substrate specificity

The substrate specificity of the enzyme was tested using a 4 mM concentration of each substrate chromogenic *4-*NPG and *4*-nitrophenyl-α-d-glucopyranoside and assayed as per "β-Glucosidase assay". The 3.5 dinitrosalycyclic acid (DNS) method as described by Adesina and Onilude^29^ was used to assay cellobiose, starch, sucrose, maltose, and glucose at a final concentration of 4 mM^36^. Enzyme activities were reported as relative activity.

#### Kinetic parameters

The kinetic parameters of the pure *β*-glucosidase were determined by measuring the enzymatic activity using *4-*NPG as the substrate at different concentrations (0, 0.66, 1.33, 1.99, 2.66, 3.32, 6.64 and 13.28 mM). Enzyme activity was determined under standard assay conditions as described above. The *K*_m_ and *v*_max_ of the purified *β*-glucosidase were calculated using the double reciprocal Lineweaver–Burk plot^37^.

## Results and discussion

### Screening of significant medium constituents for *β*-glucosidase production

*Neofusicoccum parvum* strain F7 (*N. parvum* F7) is a versatile fungus described as a plant pathogen that inhabits different trees in various areas worldwide^38^. A previous study by Singh et al.^29^ was the first to report the production of *β*-glucosidases and cellulases from this isolate. Submerged fermentation was used to determine the optimal conditions for the six parameters used for enhanced *β*-glucosidase production which provided the upper and lower levels that were applied in the Plackett–Burman design (PBD)*.* Preliminary investigations to determine the effect of six parameters (Table 1) on *β*-glucosidase production were performed using the PBD. The rows in Table 2 represent the twelve different experiments conducted revealing enzyme activities ranging from 0.02 to 1.49 U/ml across the twelve experimental runs. The p*-*values in Table 5 were used to verify the significance of each of the coefficients. The model displayed an R^2^ value of 0.86. The incubation temperature (X_3_) (*p* ≤ 0.05), incubation time (X_1_) and glycerol percentage(X_6_) (p ≤ 0.1) were all shown to significantly improve *β*-glucosidase production at a 95 and 90% confidence level (Table 5). A 90% confidence interval has been used before, however, in the medical and pharmaceutical industry, this level of confidence is not accepted^39^. Both the individual and interactive effects of these three parameters were studied further using the Box–Behnken design (BBD).

**Table 5:** Analysis of variance for six variables by Plackett–Burman design for production of *β*-glucosidases by *Neofusicoccum parvum* strain F7.

	Df	Sum squares	Mean square	F-value	P-value
Incubation time (X_1_)	1	0.35	0.35	5.12	0.0732
pH (X_2_)	1	0.20	0.20	2.85	0.1521
Incubation temperature (X_3_)	1	1.10	1.10	16.05	0.0103*
Agitation (X_4_)	1	0.02	0.02	0.29	0.6119
Casein (X_5_)	1	0.02	0.02	0.32	0.5979
Glycerol (X_6_)	1	0.34	0.34	4.92	0.0773
Residuals	5	0.34	0.07		

Significant *p*-values at *p ≤ 0.05 and *p* ≤ 0.1. Adjusted R^2^ = 0.86.

### Optimization of significant variables for *β*-glucosidase production

**Figure 1:**
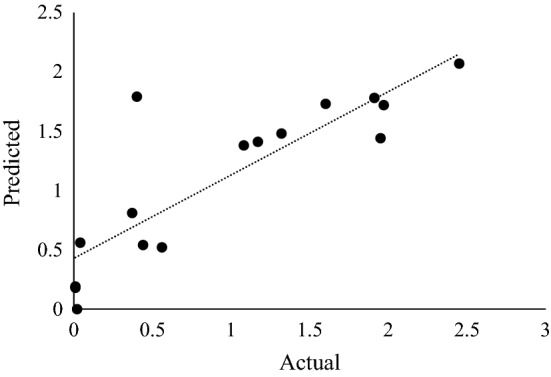
Graphical representation of the minimal difference between the actual (straight line) and predicted responses (circles) for the Response Surface Methodology design for optimal *β*-glucosidase activity.

The optimal conditions for *β*-glucosidase production by *N. parvum* F7 were determined by a 16 run BBD. The three significant variables from PBD (Table 5) were used to generate the BBD matrix and set up shake flask fermentations. The lowest activity of 0.18 U/ml was obtained in run 10 (Table 4) at the optimal incubation time (10 days), high temperature (40 °C) and low glycerol concentration (0.25%). The highest *β*-glucosidase activity of 2.51 U/ml representing a 1.5-fold increase in *β*-glucosidase activity compared to that obtained in the first attempt at optimization, i.e., one variable at a time (OVAT) experiments^29^ was obtained in run two with the following conditions: 12 days, 20 °C, and 0.5% glycerol. An F-test and T-test indicated that the increase in activity was significantly different with p-values 0.006 and 0.01, respectively. This is promising as a recent study by Nisar et al.^26^ used PBD and BBD to optimize *β*-glucosidase production by *Thermomyces dupontii* and only achieved a one-fold increase in production from 35 to 37 U/ml. Using the quadratic equation, the predicted values were determined (Table 4). The R^2^ or coefficient of determination of a valid model should be close to one. The R^2^ value was 0.83 indicating that 83% of the experimental values obtained were close to the predicted values. Although the R^2^ value was below 0.90 this is considered acceptable as a similar study by Padhiar and Modi^40^ reported R^2^ values below 0.90. Figure 1 shows that the actual response values correlate with the predicted response values, therefore the predicted *β*-glucosidase production is within the limits of the experimental factors^14^. There is an 83% chance that the model explains the measured variation in response. The corresponding response of *β*-glucosidase activity was expressed in terms of Eq. (3) using unstandardized Beta values:

3 $${\text{Y }} = {\text{ X}}_{1} + {\text{ X}}_{2} + {\text{ X}}_{3} + {\text{ X}}_{4} + {\text{ X}}_{5} + {\text{ X}}_{6}$$

$${\text{Y}} = \, - {1}.{3}0{4 } + \, 0.0{\text{57X}}_{{1}} + 0.{\text{128X}}_{{2}} - 0.0{3}0{\text{X}}_{{3}} + 0.00{\text{2X}}_{{4}} + 0.{34}0{\text{X}}_{{5}} + 0.{67}0{\text{X}}_{{6}} ,$$

### BBD

The maximum *β*-glucosidase production (2.51 U/ml) in the BBD by *N. parvum* F7 was in run two with optimal glycerol concentration (0.5%), low temperature (20 °C) and longer incubation time (12 days) (Table 4). Slightly lower activity (1.97 U/ml) was observed in run nine at the same temperature on day 10 with lower glycerol concentration (0.25%). Even lower but similar activities (1.92 U/ml) were obtained in run 6 with a 12-day incubation, optimal incubation temperature (30 °C) and lower glycerol concentration (0.25%). A study by Gao et al.^41^ reported maximal *β*-glucosidase production after 10 days of incubation. Another study by Kao et al.^1^. reported maximal *β*-glucosidase production on day eight by *Chaetomella raphigera*.

Analysis of variance (ANOVA) (Table 6) revealed that the model is significant with a p-value ≤ 0.05 whilst the lack of fit p-value is non-significant as it is greater than 0.05 this is logical and acceptable as Bezerra et al.^5^ also reported similar results. The model, the linear and square terms for incubation temperature (X_3_), and the interaction between incubation time (X_1_), incubation time and incubation temperature (X_2_) were significant in the 90–95% confidence interval with p-values of 0.04, 0.03, 0.056, and 0.02, respectively. The glycerol concentration (X_3_) displayed no significant (p-value 0.08) effect on *β*-glucosidase production. It is important to note that incubation temperature (X_2_) was the only significant factor in the 0.05 level of significance whilst glycerol concentration (X_3_) and incubation time (X_1_) were significant in the 0.1 level of significance in the PBD. Three variables are required for BBD; therefore, incubation time (X_1_) and glycerol concentration (X_3_) were considered significant, therefore this may have resulted in the two factors displaying no level of significance in the BBD. The second-order regression equation provides the *β*-glucosidase activity produced by the *N. parvum* strain F7 as a function of incubation time (X_1_), incubation temperature (X_2_), and glycerol (X_3_) which can be presented in the following Eq. (4):

4 $${\text{Y}}\, = \,{1}.{43}\, + \,0.{\text{313X}}_{{1}} \, - \,0.{\text{113X}}_{{2}} \, + \,0.0{3}0{\text{X}}_{{3}} \, + \,0.0{\text{1X}}_{{1}}^{{2}} \, - \,0.0{\text{1X}}_{{2}}^{{2}} \, + \,0.000{\text{9X}}_{{3}}^{{2}} \, - \,0.0{\text{4X}}_{{{12}}} \, + \,0.00{\text{9X}}_{{{13}}} \, + \,0.00{\text{3X}}_{{{32}}} ,$$

where Y is the peak area, X_1_ is the incubation time, X_2_ is the incubation temperature and X_3_ is the glycerol concentration. The model constants and coefficients were generated using the unstandardized beta values.

**Table 6:** Analysis of variance by the Box–Benhken design for the production of *β*-glucosidases by *Neofusicoccum parvum* strain F7.

	Estimate	Standard error	t-value	*p*-value
Model	− 13.18	5.09	− 2.59	0.04*
Incubation time (X_1_)	1.61	0.96	1.67	0.15
Incubation temperature (X_2_)	0.44	0.15	2.85	0.03*
Glycerol (X_3_)	− 1.29	4.95	− 0.26	0.80
Incubation time (X_1_): incubation temperature (X_2_)	− 0.23	0.01	− 3.11	0.02*
Incubation time (X_1_): glycerol (X_3_)	− 0.19	− 0.38	− 0.51	0.63
Incubation temperature (X_2_): glycerol (X_3_)	0.07	0.06	1.05	0.33
Incubation time (X_1_)^2^	− 0.02	0.05	− 0.52	0.62
Incubation temperature (X_2_)^2^	− 0.004	0.002	− 1.37	0.056
Glycerol (X_3_)^2^	1.30	2.97	0.44	0.68

Significant *p*-values at *p ≤ 0.05 and *p* ≤ 0.1. Adjusted R^2^ = 0.83. Lack of fit *p*-value = 0.7.

### Interaction of variables

**Figure 2:**
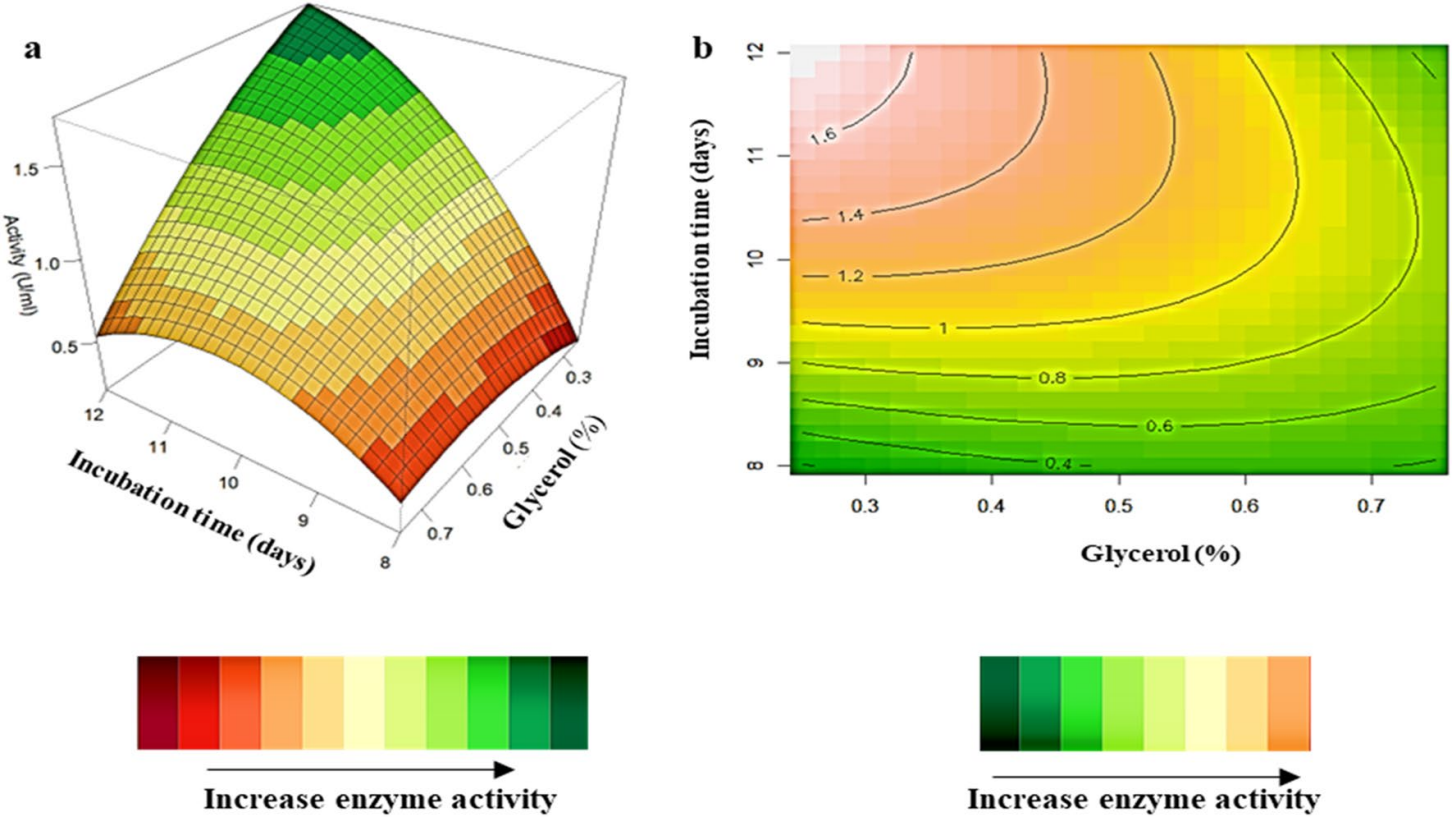
3D-response surface plots (highest activity represented by green) (**a**) and contour plots (highest activity represented by peach) (**b**) of the combined effects of incubation time (X_1_) and glycerol concentration (X_3_) on *β*-glucosidase production by *Neofusicoccum parvum* strain F7.

**Figure 3:**
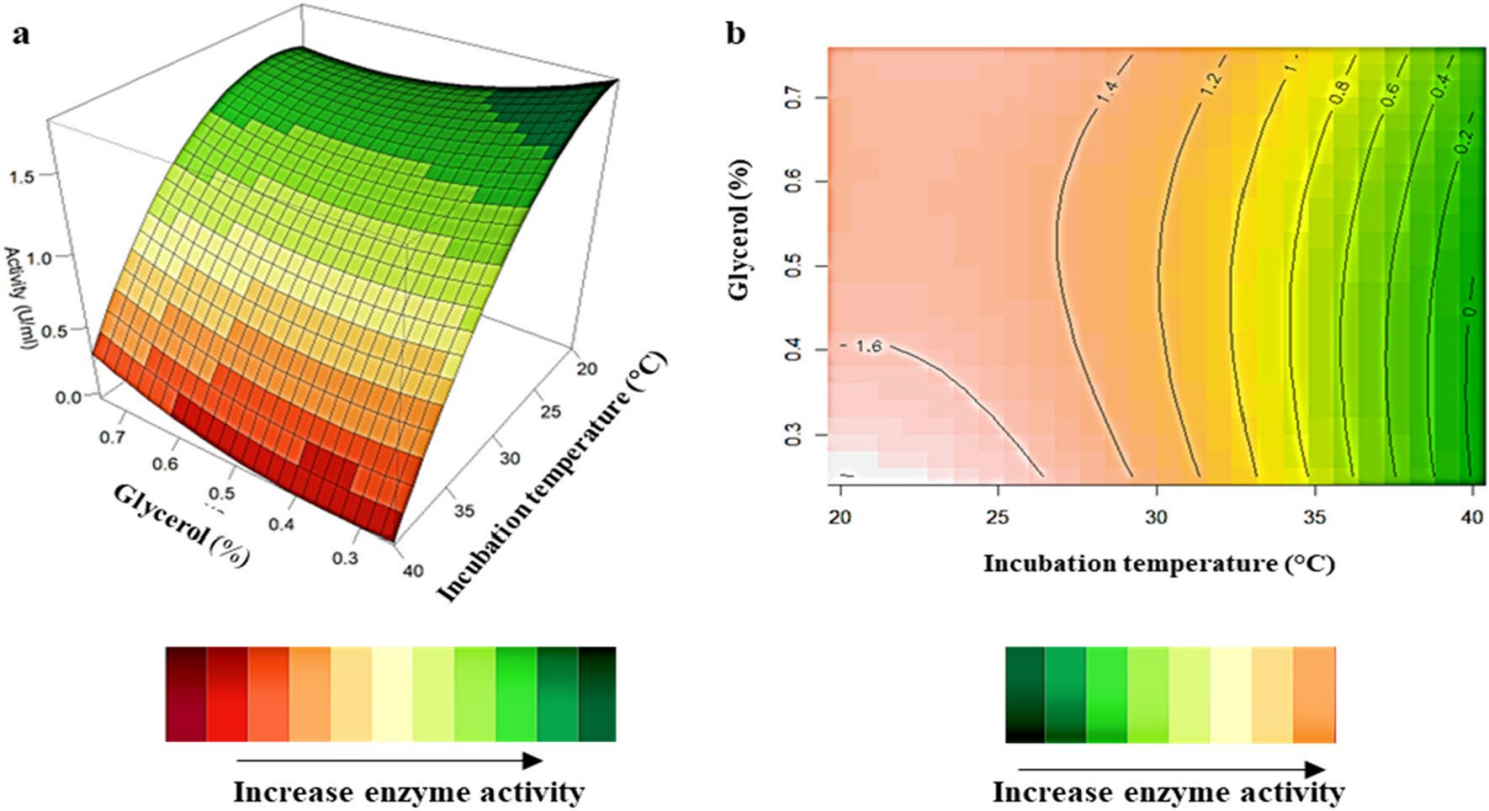
3D-response surface plots (highest activity represented by green) (**a**) and contour plots (highest activity represented by peach) (**b**) of incubation temperature (X_2_) and glycerol concentration (X_3_) on *β*-glucosidase production by *Neofusicoccum parvum* strain F7.

**Figure 4:**
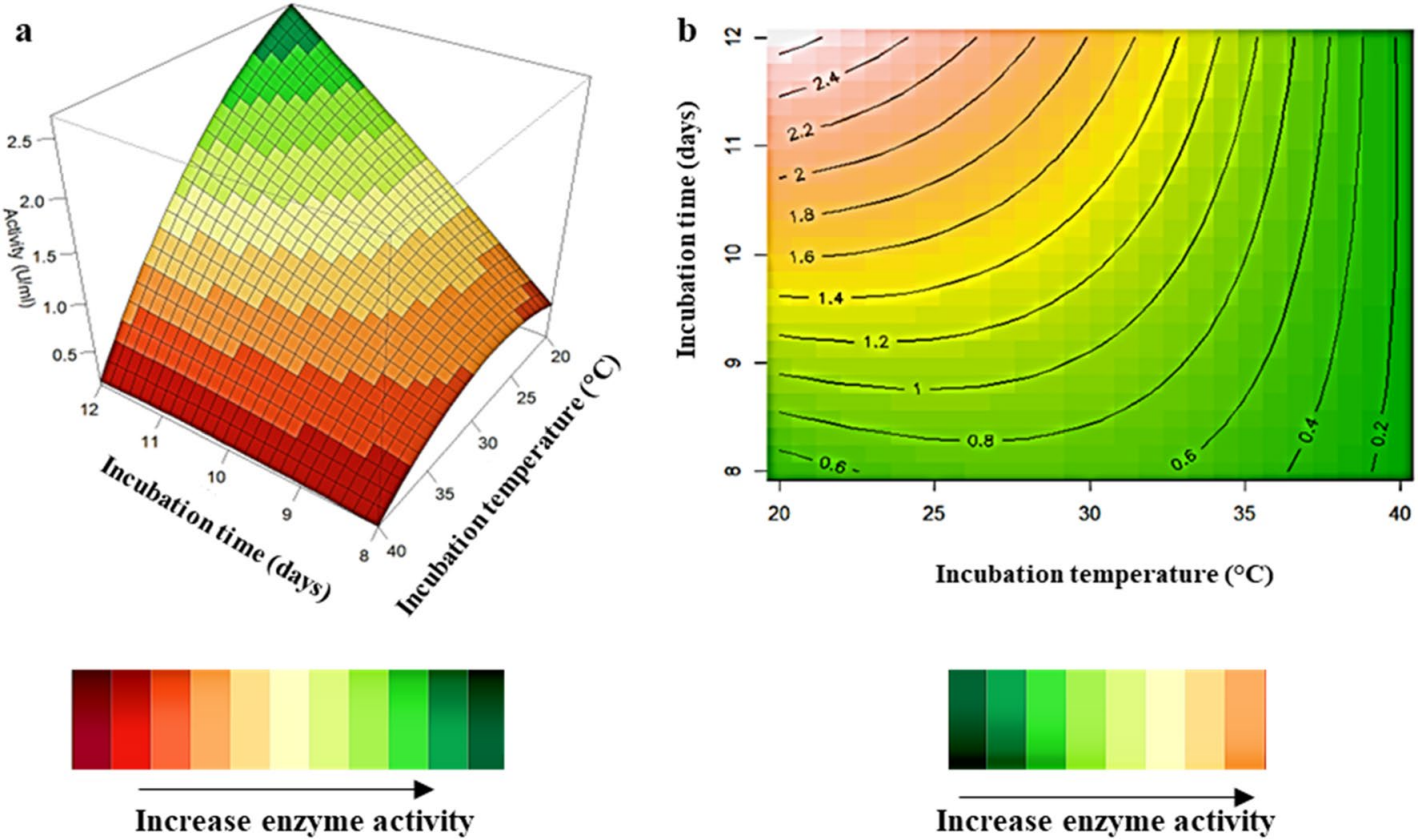
3D-response surface plots (highest activity represented by green) (**a**) and contour plots (highest activity represented by peach) (**b**) of incubation time (X_1_) and incubation temperature (X_2_) on *β*-glucosidase production by *Neofusicoccum parvum* strain F7.

The relationship between the parameters and the responses generated by the quadratic model, and the determination of the optimal level of each variable for *β*-glucosidase production was analysed by 3D response surface plots (Figs. 2, 3, 4). The z-axis in the 3D response surface plots refers to *β*-glucosidase activity versus any two variables whilst the other variables are at their optimal levels. The contour plots (Figs. 2b, 3b, 4b) display an elliptical shape indicating that all the parameters interact with each other and are dependent on one another an increase in activity is represented by the peach-shaded regions^15^. Figure 2a,b illustrates the combined effects of incubation time and glycerol concentration. *β*-Glucosidase activity is directly proportional to time, activity increases with time at all the glycerol concentrations tested. Figure 2b illustrates the contour plot, high enzyme activity was obtained at the longest incubation period (12 days) and at glycerol concentrations between 0.3 and 0.5%. Mahapatra et al.^29^ also obtained high *β*-glucosidase production from a *Proteus mirabilis* strain at longer incubation times. Figure 3a,b shows that the highest *β*-glucosidase production occurs at low temperatures (20 °C) and a wide range of glycerol concentrations (0.3–0.7%). The 3D-response surface plots (Figs. 2a, 3a) show that the highest *β*-glucosidase activity was obtained at low temperature (20 °C), 0.3% glycerol and longer incubation time (12 days). Previous studies using the OVAT optimization approach obtained high *β*-glucosidase production at 20 °C and 30 °C with optimal enzyme production at 30 °C^44^. Studies by El-Naggar et al.^17^ and Job et al.^27^ obtained the highest *β*-glucosidase production at low temperatures of 25 and 28 °C, respectively. Carbon concentration also displayed no significant effect in these studies. Figure 4a,b illustrates that incubation time and temperature are inversely proportional to each other the highest *β*-glucosidase activity was obtained at longer incubation times (12 days) and lower temperatures (20 °C). The 3D plots and the contour plots correlate with the results of the ANOVA table which indicate that the interactions between incubation time and temperature are significant with a p-value ≤ 0.05. These results are corroborated by several other studies that reported higher *β*-glucosidase production at longer incubation times and lower incubation temperatures^17,27,29^. The interaction between incubation time and temperature (2.51 U/ml) had the highest effect on *β*-glucosidase production compared to the other two interactions between incubation time and glycerol concentration (1.5 U/ml) and incubation temperature and glycerol concentration (1.5 U/ml). This study demonstrated a notable increase in *β*-glucosidase activity using the statistically designed experiments when compared to OVAT experiments^29^.

### Scaled-up fermentation in optimized conditions

Two 2 L flasks containing 200 ml of the optimal medium (minimal media pH 6.0) supplemented with 0.3 and 0.4% glycerol, respectively were incubated at optimal conditions (20 °C for 12 days shaking at 175 rpm) determined using the polynomial equation together with the contour and response surface 3D-plots. *β*-Glucosidase activity of 2.01 U/ml was obtained for both glycerol concentrations confirming that the concentration of glycerol had no significant effect, however, compared to the smaller scale production a decrease of 0.5 U/ml was observed. This observation is not unusual as a reduction in enzyme activity during the scaling up of enzyme production is a common challenge experienced by researchers, especially during scale-up from shake flask fermentations to bioreactor fermentations. However, in this instance, the scale-up was in reaction vessels of the same design. We attribute the difference to a possibly lower biomass in the larger vessels as the inocula were not standardised using spore counts but mycelial plugs. A small difference when one plug is used would become exaggerated when several are applied to the larger volume vessel.

### Purification of the *N. parvum β*-glucosidase, PAGE and zymography

Ammonium sulphate precipitation, dialysis, and chromatographic methods were combined to purify the β-glucosidase enzyme from *N. parvum* F7 successfully. Out of the ten dialysed fractions, the 80, 90 and 100% fractions displayed the highest protein precipitation and *β*-glucosidase activities (Table 7). The dialysis buffer was also assayed for *β*-glucosidase activity of which no activity was detected. These fractions were combined for further purification and analysis. Ahmed et al.^3^, Cao et al*.*^9^, Zhang et al.^25^, Narasimha et al.^35^, and Christakopoulos et al.^42^, reported that microbial *β*-glucosidases were precipitated using ammonium sulphate saturation of 75%, 80%, 80% and 90%, respectively with the 80% saturation precipitating highest quantities of *β*-glucosidases with a 39.6 and 63% yield, respectively. The combined fractions recovered 35% of the protein whilst 44% was retained in the supernatant, and the remainder was precipitated in the other seven ammonium sulphate fractions (Table 7). Although a large amount of protein was in the supernatant only 0.48% of *β*-glucosidase activity was present, therefore, no further precipitation was carried out. The specific activity of the combined fractions decreased from 11.6 to 9.1 U/ml which may be due to loss of enzyme stability during overnight incubation. Loss of stability may be attributed to a change in temperature and the stirring speed of the ammonium sulphate solution^3^.

**Table 7:** Purification table for *β*-glucosidase from *Neofusicoccum parvum* strain F7.

	Total protein (mg)	Total activity (U)	Specific activity (U/mg)	Yield (%)	Fold purity
Crude extract	90	1042.30	11.58	100	1
Ammonium sulphate fraction					
Combined 80%, 90%, 100%	31.90	288.70	9.05	27.7	2.20
Supernatant	40	5	0.13	0.48	0.01
Anion exchange (HiTrap Q FF)					
Combined ammonium sulphate fraction	4.20	125.13	29.79	12.01	2.57
Gel extraction					
Bgl1	1	21.80	21.8	2.09	2.42
Bgl2	0.60	10.80	18	1.04	4.33
Bgl3	0.20	76.80	384	7.37	33.22

**Figure 5:**
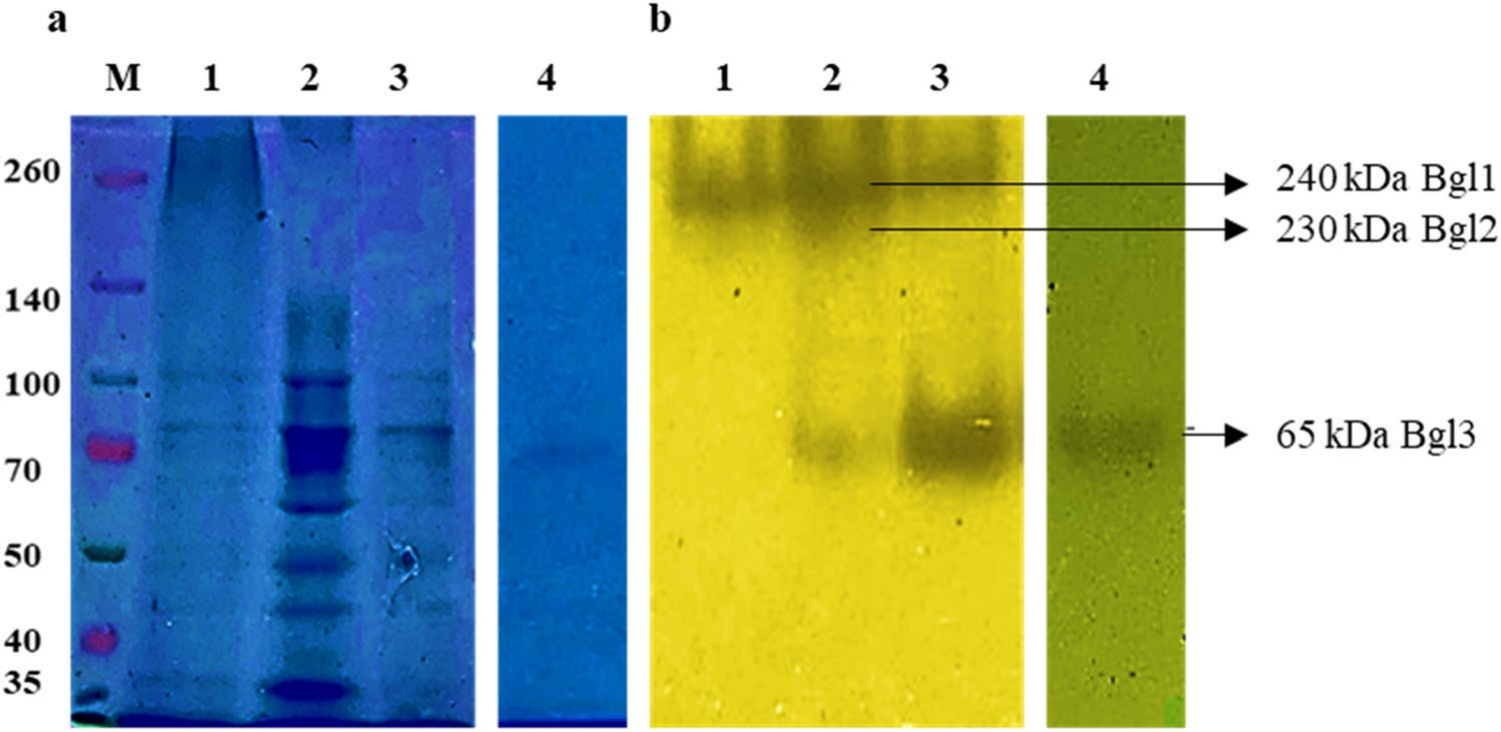
12% SDS PAGE (**a**) Lanes M: Molecular mass marker (Thermo scientific, USA), 1: crude enzyme extracts, 2: 80% ammonium sulphate fraction, 3: anion exchange fraction, 4: purified *β*-glucosidase, 8% Native PAGE (**b**) displaying zones of black precipitation 1: crude enzyme extract, 2: combined ammonium sulphate fractions, 3: anion exchange fraction and 4: purified *β*-glucosidase. The four original gels are presented in Supplementary Figs. 1–4.

The dialysed combined fractions were loaded onto a DEAE Sephadex column for further purification. A 0–2 M sodium chloride concentration gradient was used to elute the bound protein. *β*-Glucosidase activity was determined for both the bound and unbound protein fractions. The primary peak eluted at 500 mM sodium chloride and the corresponding fraction displayed a specific activity of 29.8 U/mg, and a 2.57-fold purity. After anion exchange chromatography SDS-PAGE confirmed that there was more than one band present in the fraction. After anion exchange chromatography the total protein (4.20 mg) and fold purity (12%) decreased, the specific activity and fold purity increased to 29.79 U/mg and 2.57, respectively. Qin et al*.*^11^ also observed similar results in their attempts to purify *β*-glucosidases from *F. chlyamydosporum*. Size exclusion chromatography with a 250 kDa molecular cut-off was carried out with 50 mM Tris (pH 8.0) buffer, however, there was no protein or *β*-glucosidase activity detected in any of the eluted fractions. The anion exchange fraction was then loaded onto a Native PAGE gel which consisted of an 8% resolving gel and 4% stacking gel for enzyme extraction from the gel after electrophoresis. Electrophoresis was carried out at 50 V for 3 h. After electrophoresis and activity staining, active *β*-glucosidase protein bands were detected by black precipitates. Figure 5b lane two showed three zones of black precipitation indicating the presence of three *β*-glucosidase isoforms: Bgl1 (240 kDa) displaying a bright band and two faint bands representing, Bgl2 (230 kDa) and Blg3 (65 kDa). This observation implies that the interactions among the three tested variables at three different levels in the BBD indicate the presence of more than one *β*-glucosidase enzyme or isoform present in the crude extract. Based on Table 4 and Fig. 5b there are three *β*-glucosidase isoforms in the crude extracts as three bands appear on the native PAGE gel and high *β*-glucosidase activities were observed in runs two, six and nine. Isoforms are produced at different periods of incubation, various types and concentrations of carbon and different temperatures for maximal *β*-glucosidase activity^43^. Qin et al.^11^ reported that two *β*-glucosidase isoforms were produced by *Fusarium chlamydosporum* (*F. chlamydosporum*) after four days at 4 °C in solid substrate fermentation. Different forms of *β*-glucosidases differ in stability, catalytic efficiency, absorption, and activity on various substrates^26^. Various alleles of the same gene, variable mRNA processing, proteolytic digestion, and post-translational modifications are variables that influence the production of multiple forms of *β*-glucosidases^3,45^. Srivastava et al.^44^ stated that multiplicity is a common phenomenon for *β* glucosidases. This phenomenon is beneficial as the enzymes from crude extracts can be purified and applied in different industries according to their characteristics^22^.

Each band was cut off and ground in a pre-cooled mortar and pestle. The samples were leached with 50 mM sodium phosphate buffer (pH 6.0) at 4 °C for 12 h and centrifuged at 14,000×*g* for 15 min at 4 °C. The supernatants were removed and dialysed in the same buffer overnight at 4 °C. The dialysed samples were then concentrated in a 3 kDa spin column and the specific activities were determined to be 21.8, 18 and 384 U/mg (Table 7) for Bgl1, Bgl2 and Bgl3, respectively. Bgl3 displayed the highest purification fold (33.22), *β*-glucosidase yield (7.37%), and specific activity (384 U/mg). Followed by Bgl2 with a 4.33 purification fold and the lowest *β*-glucosidase yield of 1.04% and Bgl1 which displayed a 2.42 purification fold and 2.09% yield. Crude enzyme extracts contain various enzymes with different concentrations Rani et al.^46^ reported that *β*-glucosidase enzymes in crude extracts are present at low titres as compared to other enzymes and display isoforms. Due to the above the majority of the protein present in the crude extract was not responsible for *β*-glucosidase activity, therefore, the supernatant displayed a high protein content with low *β*-glucosidase activity. The *β*-glucosidase activity obtained in the crude extract represents the combined activities of all three isoforms as seen in Fig. 5. After purification, it was observed that each isoform was present at different concentrations as different yields were obtained for each (Table 7). Rani et al.^14^ further explained that *β*-glucosidase isoforms displaying lower titres are more likely to display tolerance to glucose compared to those at higher titres. To identify the *β*-glucosidase enzyme with the highest tolerance to glucose the IC_50_ values were determined.

### Characterization of glucose tolerant *β*-glucosidase (Bgl3)

#### Glucose tolerance of β-glucosidase

**Figure 6:**
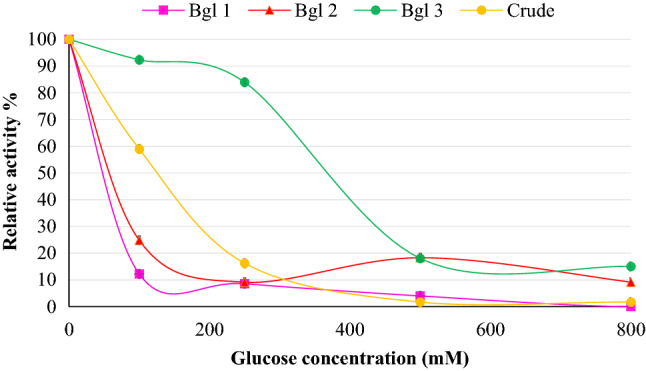
Glucose tolerance of the crude *Neofusicoccum parvum* strain F7 extract (119.2 mM), Bgl1 (2.6 mM), Bgl2 (22.6 mM) and Bgl3 (319.5 mM). The data points represent the mean ± SD (n = 2).

The IC_50_ values for the crude extract, Bgl1, Bgl2, and Bgl3 were found to be 119.2 mM, 2.6 mM, 22.6 mM, and 319.5 mM, respectively (Fig. 6). According to the classification of *β*-glucosidases, a *β*-glucosidase enzyme may be classified as glucose tolerant if the IC_50_ value is greater than 100 mM and those exhibiting IC_50_ values less than 100 mM are classified as *β*-glucosidases that are strongly inhibited by low glucose concentrations^24^. Therefore, Bgl1 and Bgl2 were strongly inhibited by low glucose concentrations whilst Bgl3 demonstrated glucose tolerant characteristics with an IC_50_ of 319.5 mM. The tolerance to glucose observed in the crude extract is lower than that of Bgl3 indicating that the isoform’s tolerance to glucose increased in its pure state. This may be attributed to a higher concentration of Bgl3 after purification^2^. The *β*-glucosidase from *Exiguobacterium antarcticum* and *Alicyclobacillus* sp. A4 displayed IC_50_ values for glucose of 1 M^21^ and 800 mM respectively^9^. The Bgl3 isoform in this study exhibited a higher tolerance to glucose compared to the *β*-glucosidase enzyme obtained from *Aureobasidium pullulans* in a study by Baffi et al.^47^ that showed an IC_50_ of 50 mM. Although multiple different *β*-glucosidase enzymes revealed IC_50_ values higher than that obtained in the current study it is important to study the characteristics and prospects for applications of the enzyme as it may be optimally applied to supplement current commercial cellulolytic cocktails^34^. Bgl3 demonstrated the highest tolerance to glucose and was, therefore, taken forward for further characterization. The purified enzyme presented a molecular mass of 65 kDa. *β*-Glucosidases are classified based on their origin, molecular mass, catalytic reactions, and amino acid sequence similarities. *β*-Glucosidases belong to GH families I, III, V, IX, 30 and 116. GH III *β*-glucosidases originate from fungi and display molecular masses of 65–90 kDa. Therefore, based on these two factors we can infer that the Bgl3 enzyme belongs to the GH III family^2^. The IC_50_ value of 319.5 mM observed for Bgl3 is noteworthy as Cao et al.^9^ reported that in several studies IC_50_ values below 100 mM were observed for GH III family *β*-glucosidases due to feedback inhibition of enzyme activity by glucose.

#### Optimum pH, temperature, and stability of Bgl3

**Figure 7:**
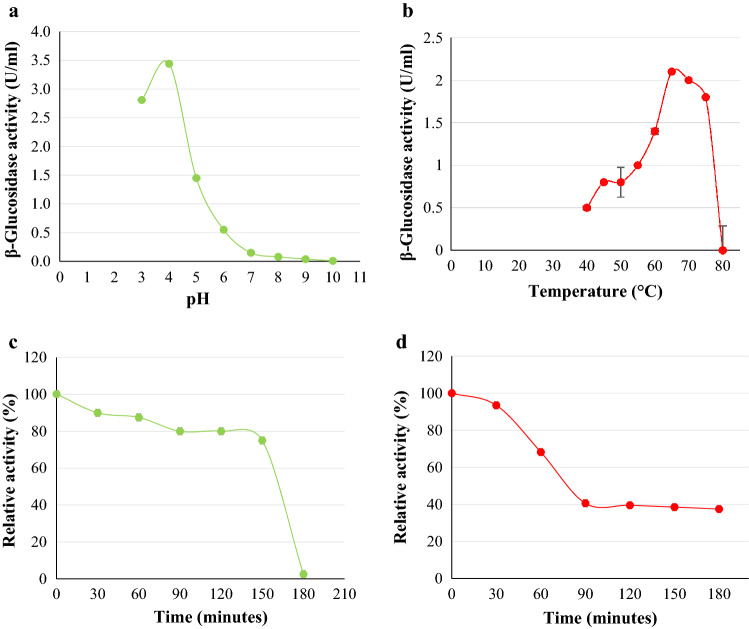
pH (**a**) and temperature (**b**) optima and pH stability (pH 4.0) (**c**) and thermostability (65 °C) (**d**) of Bgl3 produced by *Neofusicoccum parvum* strain F7. Data points represent the mean ± SD (n = 2).

The purified Bgl3 *β*-glucosidase from *N. parvum* F7 displayed optimum activity at pH 4.0 and temperature 65 °C (Fig. 7) indicating that the enzyme is both thermophilic and able to withstand acidic environments^48^. Qin et al.^11^ reported optimal *β*-glucosidase activities at 60 °C for *F. chlamydosporum* and pH 4.0 for *F. moniliforme*. The Bgl3 was stable at pH 4.0, retaining 80% activity for 120 min. After 150 min, less than 5% of activity was lost, however, a drastic decline in enzyme activity was observed with all activity lost between 151 and 180 min. The enzyme lost 60% activity at 65 °C in the first hour, thereafter a decline to 40% residual activity was observed, after which the enzyme remained stable for another 90 min. Karami et al.^49^ reported stability of *β*-glucosidase for one hour at 70 °C with 95% retention in activity. Bgl3 displayed stability for three hours retaining 40% activity at 65 °C. *β*-glucosidases obtained from *Caldicellulosiruptor saccharolyticus* displayed similar optimal enzyme activities and stability in a pH 5.0 buffer between 70 and 80 °C. Enzymes that prefer higher temperatures, display stability, and can withstand acidic environments are advantageous when searching for enzymes for industrial applications^49,50^.

#### Effect of metal ions on the purified Bgl3 activity

The effects of eight metal ions (Ca^2+^, Co^2+^, Fe^2+^, Mg^2+^, Mn^2+^, Zn^2+^, K^+^, and Na^+^) on *β*-glucosidase activity were determined at a final concentration of 4 mM at the optimal pH and temperature (4.0 and 65 °C) (Table 8). *β*-Glucosidases were either inhibited or activated by the metal ions^18^. All eight metal ions reduced *β*-glucosidase activity in Bgl3 by 50% or more in this study. These findings were also reported by Qin et al.^11^. The highest *β*-glucosidase activity was in the presence of FeSO_4,_ and the lowest activity was in the presence of ZnSO_4,_ CoCl_2,_ KH_2_PO_4_ NaCl, similar results were also reported in other studies also where *β*-glucosidase activity declined in the presence of MnSO_4,_ ZnSO_4_ and CoCl_2_^11,18^.

**Table 8:** Effect of metal ions on purified Bgl3 by *Neofusicoccum parvum* strain F7 (relative activity). Each data point represents mean ± SD (n = 2).

Metal ion (4 mM)	Relative activity (%)
Control	100.00 ± 0.01
FeSO_4_	45.44 ± 0.01
MgSO_4_	38.02 ± 0.01
ZnSO_4_	33.33 ± 0.01
CoCl_2_	31.38 ± 0.01
CaCl_2_	35.42 ± 0.01
NaCl	34.64 ± 0.01
KH_2_PO_4_	34.11 ± 0.01
MnSO_4_	39.71 ± 0.01

#### Substrate specificity of the purified enzyme

**Figure 8:**
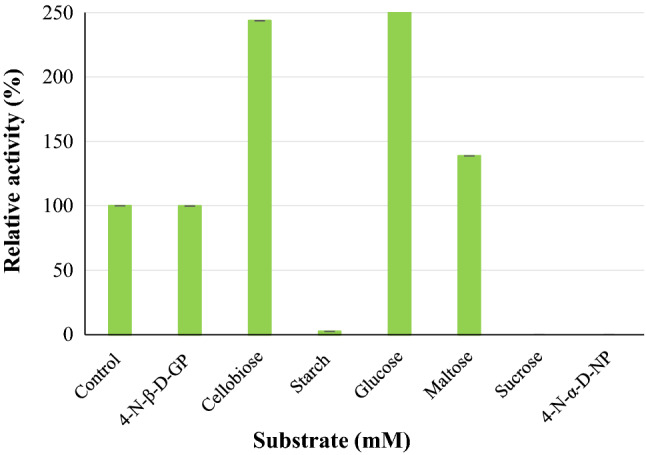
Substrate (4 mM) specificity of the purified Bgl3 produced by *Neofusicoccum parvum* strain F7. Each data point represents mean ± SD (n = 2).

To determine the substrate specificity of the purified *β*-glucosidase the following substrates were tested at a concentration of 4 mM under optimal conditions (50 mM sodium acetate pH 4.0 and 65 °C) *4*-nitrophenyl-*β*-d-glucopyranoside, *4*-nitrophenyl-α-d-glucopyranoside, cellobiose, starch, glucose, maltose and carboxymethylcellulose. Higher hydrolytic activity was seen for maltose (139%), Glucose (259%), and cellobiose (244%) compared to carboxymethylcellulose and starch (Fig. 8). The purified *β*-glucosidase exclusively hydrolysed *4*-NPG, disaccharide, and oligosaccharide sugars. *β*-Glucosidases are divided into three groups based on substrate specificity^51^. Group one is known as aryl-*β*-glucosidases as they display hydrolytic activity towards aryl-*β*-glucose, the second group consists of cellobiases that only hydrolyze oligosaccharides and the third group consists of broad specificity *β*-glucosidases that display hydrolytic activity on a broad range of substrates^7^. The purified *β*-glucosidase in this study displayed activity towards majority of the substrates tested which included both oligosaccharides and disaccharides. This indicates that Bgl3 belongs to the third group of *β*-glucosidases^7^. Bgl3 exhibited no activity when sucrose was used as a substrate, this may be attributed to the α-configuration of glucose in sucrose^11^. Bgl3 activity is enhanced by 144–244% when cellobiose is used as a substrate this indicates that the enzyme shows great potential for application in cellulose hydrolysis as *β*-glucosidase enzymes are responsible for the breakdown of cellobiose to glucose monomers.

### Kinetic analysis

**Figure 9:**
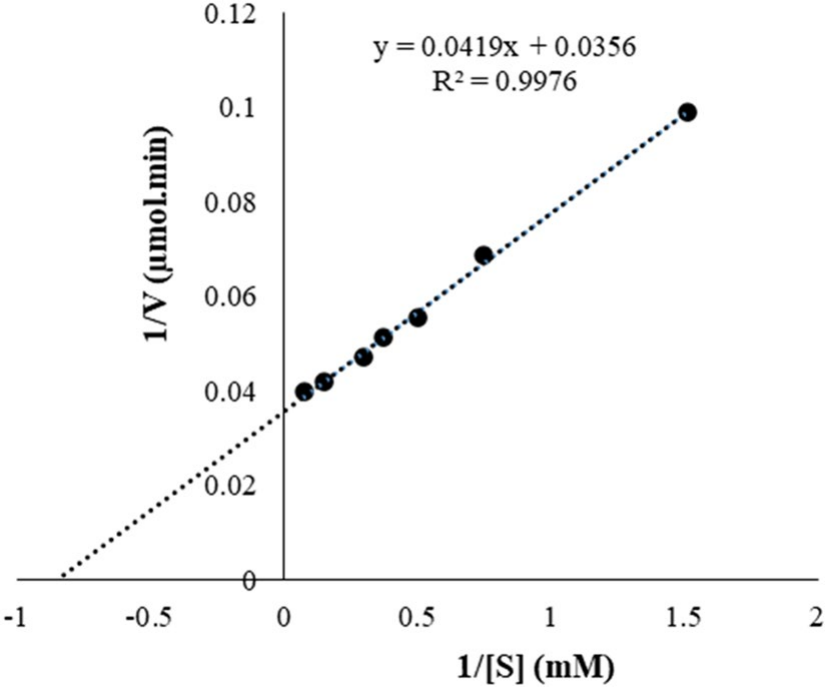
Lineweaver–burk plot of the activity of the purified Bgl3 from *Neofusicoccum. parvum* strain F7 on *4*-nitrohenyl-*β*-d-glucopyranoside. Data points represent the mean ± SD (n = 2).

The Michaelis constant, *K*_m_ was determined by measuring the substrate concentration at half the maximum velocity (Fig. 9). The concentration range of the *4*-NPG substrate under investigation was 0–13.28 mM. The study revealed *K*_m_ and *v*_max_ of 1.18 mM and 28.08 µmol/min, respectively. The value of *K*_m_ is within the range of fungal *β*-glucosidases as Qin et al.^11^ obtained a *K*_m_ value of 2.76 mM for *β*-glucosidases from *F. chlamydosporum*. The low *K*_m_ and high *v*_max_ displayed by the Bgl3 *β*-glucosidase indicate that the enzyme has a high affinity for *4*-NPG^23^^.^

## Conclusion

This study successfully optimised the production of a glucose-tolerant *β*-glucosidase via statistical modelling using Plackett–Burman and Box–Behnken design in submerged fermentation. The most influential independent variables were identified and optimized, resulting in a 36% increase in *β*-glucosidase production. The *Neofusicoccum parvum* strain F7 crude extract contained three *β*-glucosidase isoforms. Bgl3 was successfully purified with a 7.37% *β*-glucosidase yield, 33.22 fold purity and exhibited the highest tolerance to glucose, as well as an acidic and thermophilic nature with a specific activity of 384 U/mg. *β*-Glucosidases are applied in multiple industries that include the paper, animal feed and flavour industries. The increase in hydrolytic activity towards cellobiose indicates that Bgl3 would be advantageous in the paper industry for cellulose hydrolysis. Future studies will include supplementing a commercial and crude cellulolytic cocktail with purified Bgl3 to determine the effect of external *β*-glucosidase supplementation on cellulose hydrolysis. If the effects are favourable further studies should include cloning and overexpressing the Bgl3 isoform to produce meaningful levels for industrial application.

## Supplementary Information

Supplementary Figures.

## Data Availability

The datasets used and/or analysed during the current study are available from the corresponding author upon reasonable request. Other data generated or analysed during this study are included in this article [and its supplementary information file].
