# Supplementary material for: A glucose tolerant β-glucosidase from a newly isolated Neofusicoccum parvum strain F7: production, purification, and characterization
Source: Sci Rep. 2023 Mar 29;13:5134. doi: 10.1038/s41598-023-32353-6 (PMC10060427; doi:10.1038/s41598-023-32353-6)
Supplement: Supplementary file 1 — Supplementary Figures. [file 41598_2023_32353_MOESM1_ESM.docx]

**M 1 2 3**


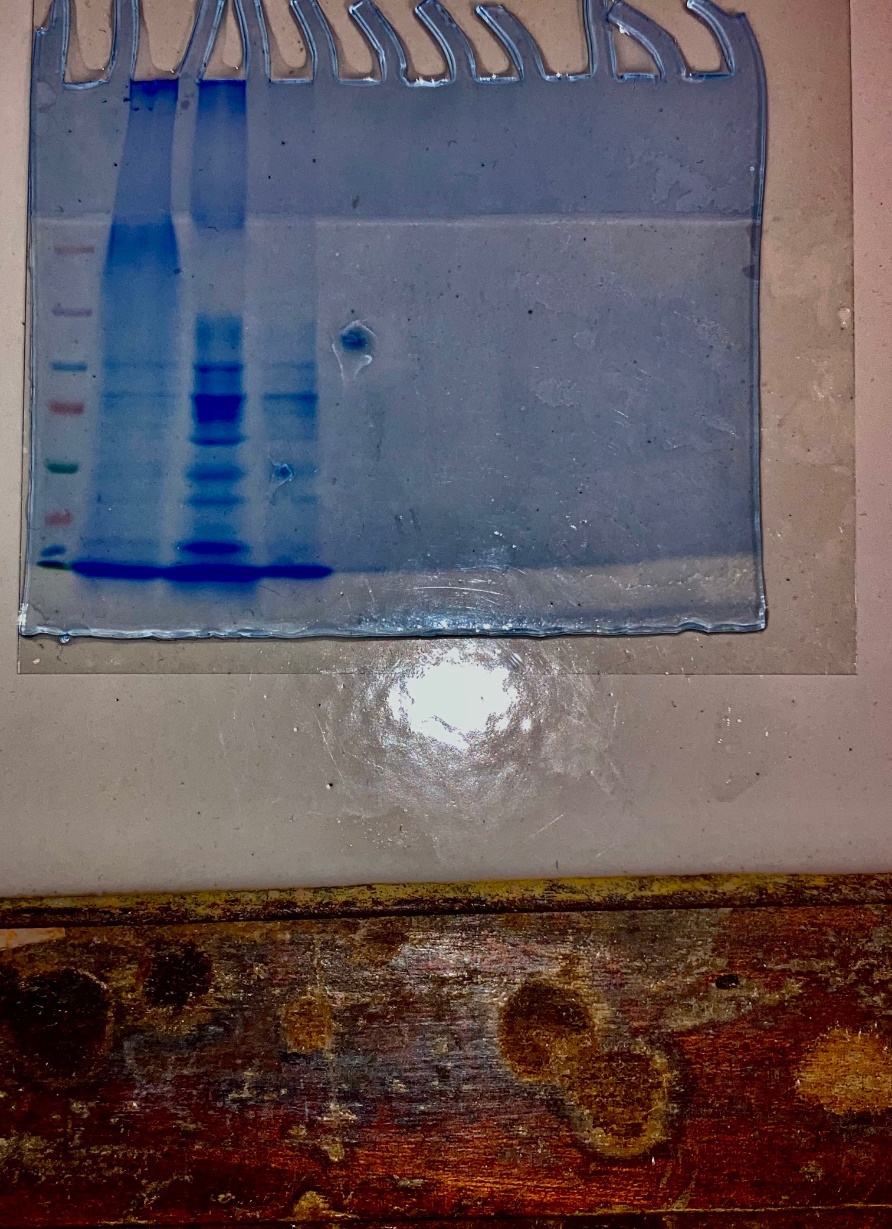


Supplementary Figure 1: Original 12% SDS PAGE gel image Lanes M: Molecular weight marker (Thermo scientific, USA), 1: crude enzyme extract, 2: 80% ammonium sulphate fraction, 3: anion exchange fraction of *β*-glucosidase production from *Neofusicoccum parvum* F7. Lanes M, 1, 2, and 3 were cropped for the manuscriot and are represented in Figure 5a, Lanes M, 1, 2 and 3.

**1 M**


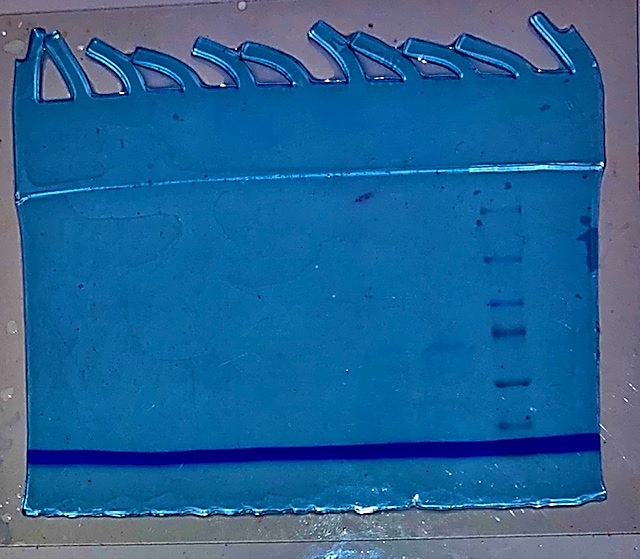


Supplementary Figure 2: Original 12% SDS PAGE gel image Lanes M: Molecular weight marker (Thermo scientific, USA), 1: purified Bgl3 from the crude extract of *Neofusicoccum parvum* F7. Lane 1 was cropped for the manuscript and is represented in Figure 5a, lane 4.

**a 1 2 3 4 5 6 b M**


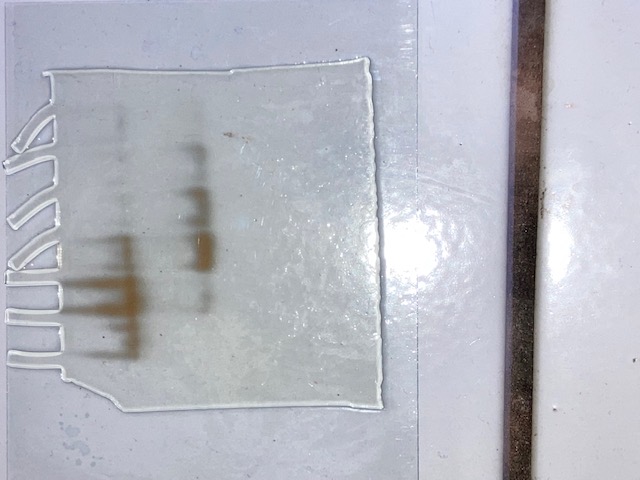

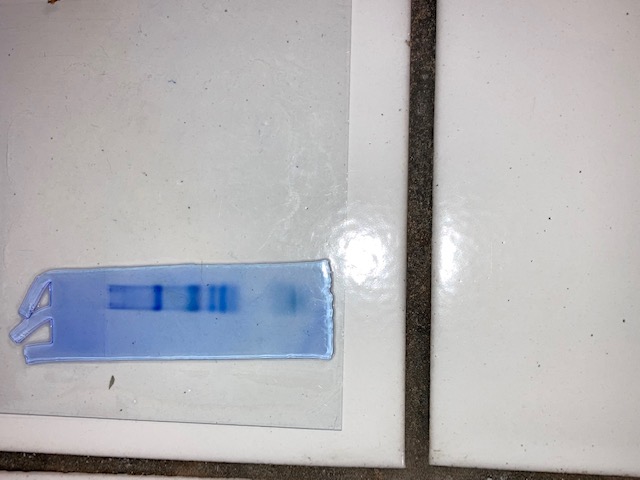


Supplementary Figure 3: Original 8% Native PAGE gel image displaying zones of black precipitation (a) Lanes 1: crude enzyme extract, 2: combined ammonium sulphate fractions, 3: anion exchange fraction of concentrated samples and 4 crude enzyme extract, 5 combined ammonium sulphate fractions, and 6 anion exchange fractions of unconcentrated samples and 4: purified *β*-glucosidase. Lanes 1, 2, and 3 were cropped from this gel for the manuscript and is represented in Figure 5b as lanes 1,2 and 3. (b) Lane M molecular weight marker (Thermo scientific, USA) stained with Coomassie Brilliant blue.

**a 1 2 3 b M**


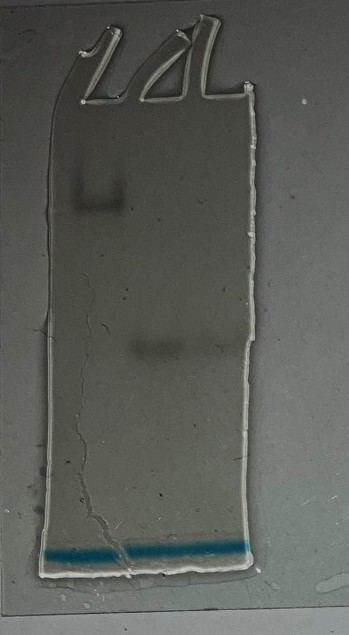

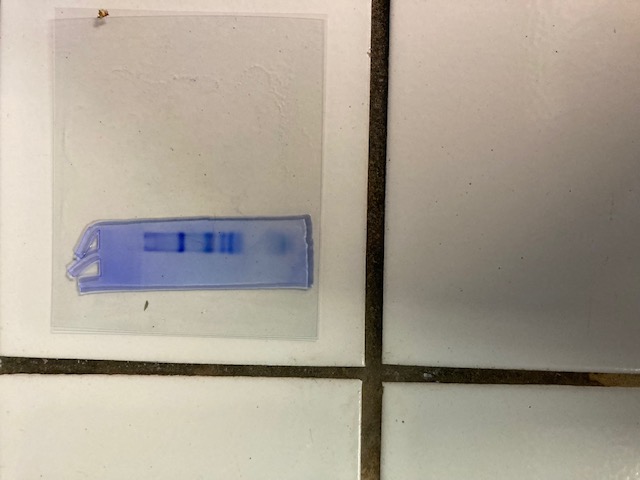


Supplementary Figure 4: Original 8% Native PAGE gel image displaying zones of blackening of purified Bgl3 from *Neofusicoccum parvum* F7. (a) Lane 1 crude unconcentrated sample lane 2 and 3 purified Bgl3. Lanes 2 from this gel was cropped for the manuscript and is represented in Figure 5b. (b) Lane M molecular weight marker (Thermo scientific, USA) stained with Coomassie Brilliant blue.
